# Safety of Sodium Zirconium Cyclosilicate in Critically Ill Patients With Impaired Gastric Motility: A Case of Persistent Intragastric Precipitation

**DOI:** 10.7759/cureus.93726

**Published:** 2025-10-02

**Authors:** Shinya Yata, Yudai Takatani, Yuki Sato, Tomohiro Terada, Shigeru Ohtsuru

**Affiliations:** 1 Department of Primary Care and Emergency Medicine, Kyoto University Graduate School of Medicine, Kyoto, JPN; 2 Department of Clinical Pharmacology and Therapeutics, Kyoto University Hospital, Kyoto, JPN

**Keywords:** critically ill patients, gastroparesis, hyperkalemia, intragastric precipitation, sodium zirconium cyclosilicate

## Abstract

Sodium zirconium cyclosilicate (SZC) is a novel potassium-binding agent used to treat hyperkalemia. Although SZC is generally considered safe, its efficacy and safety in critically ill patients remain unclear.

We report a novel adverse event of persistent intragastric SZC precipitation in a critically ill patient. A man in his 20s was admitted after a cardiac arrest and developed hyperkalemia and renal dysfunction. Following four days of SZC administration via a nasogastric tube, chest radiography revealed a high-density intragastric mass. Gastric lavage and Gastrografin administration confirmed the presence of the precipitate and revealed severely impaired gastric motility. This case highlights the potential risks of SZC use in critically ill patients with impaired gastric motility. Factors contributing to precipitation include decreased bowel peristalsis due to intestinal ischemia and the use of sedatives and vasopressors. Close radiographic monitoring of SZC transit is recommended for early detection. Further research is required to establish optimal management strategies for such adverse events.

## Introduction

Sodium zirconium cyclosilicate (SZC) is a relatively new potassium-binding agent that lowers the serum potassium level by selectively capturing potassium ions. Hyperkalemia in critically ill patients presents an immediate life threat because of its potential to induce fatal cardiac arrhythmias, necessitating rapid and effective therapeutic interventions. SZC has become an attractive option for management due to its highly selective binding profile, rapid onset of action, and favorable tolerability compared to older, non-selective agents. Its mechanism of action involves exchanging potassium ions for hydrogen and sodium ions in the gastrointestinal tract, primarily the upper tract, providing a more rapid onset than older agents. Compared with conventional calcium polystyrene sulfonate and sodium polystyrene sulfonate, SZC is characterized by less expansion in the intestine. This property of SZC has been associated with some reported adverse events such as hypokalemia, edema, constipation, nausea, and mild gastrointestinal symptoms [1]. However, serious side effects, such as gastrointestinal perforation, are rare, and SZC is considered to be highly safe [2,3]. However, its efficacy and safety in critically ill patients remain unclear. To the best of our knowledge, no prior reports have described intragastric precipitation and retention of SZC in critically ill patients.

This case highlights the potential risk of SZC use in those with markedly reduced gastric motility, a condition frequently observed in critical illness. Herein, we report a case in which precipitate formation occurred in the stomach and the substance remained after SZC administration via a nasogastric tube in the emergency and intensive care unit (EICU).

## Case presentation

A man in his 20s was found in a state of cardiopulmonary arrest due to hanging on day X and was brought to our hospital via emergency transport. Cardiopulmonary resuscitation was continued, and spontaneous circulation was restored, 8 min after arrival at the hospital, through adrenaline administration. Blood tests on admission revealed a marked progression of acidosis and elevated liver enzyme levels (Table 1).

**Table 1:** Laboratory findings on arrival AST: aspartate aminotransferase; ALT: alanine transaminase; LDH: lactate dehydrogenase; Cre: creatinine; eGFR: estimated glomerular filtration rate; BUN: blood urea nitrogen; pCO2: partial pressure of carbon dioxide; pO2: partial pressure of oxygen; HCO3-: bicarbonate

Parameter	Value	Normal range	Unit
WBC	4,180	3,300 – 8,600	/μL
CRP	0.1	≤0.14	mg/dL
AST	544	13 – 30	IU/L
ALT	749	7 – 23	IU/L
LDH	1337	124 – 222	IU/L
Total bilirubin	0.3	0.4 – 1.5	mg/dL
Cre	1.1	0.46 – 0.79	mg/dL
eGFR	68.6	≤60	mL/min/1.73 m²
BUN	14	8 – 20	mg/dL
Na	143	138 – 145	mEq/L
K	5.6	3.6 – 4.8	mEq/L
Cl	102	101–108	mEq/L
pH	6.83	7.35 – 7.45	
pCO2	93.6	35 – 45	mmHg
pO2	37.2	80 – 100	mmHg
Lactate	17.0	≤2.0	mmol/L
Base excess	-19.7	−2 - +2	mmol/L
HCO3-	15.7	22 – 26	mmol/L

**Figure 1:**
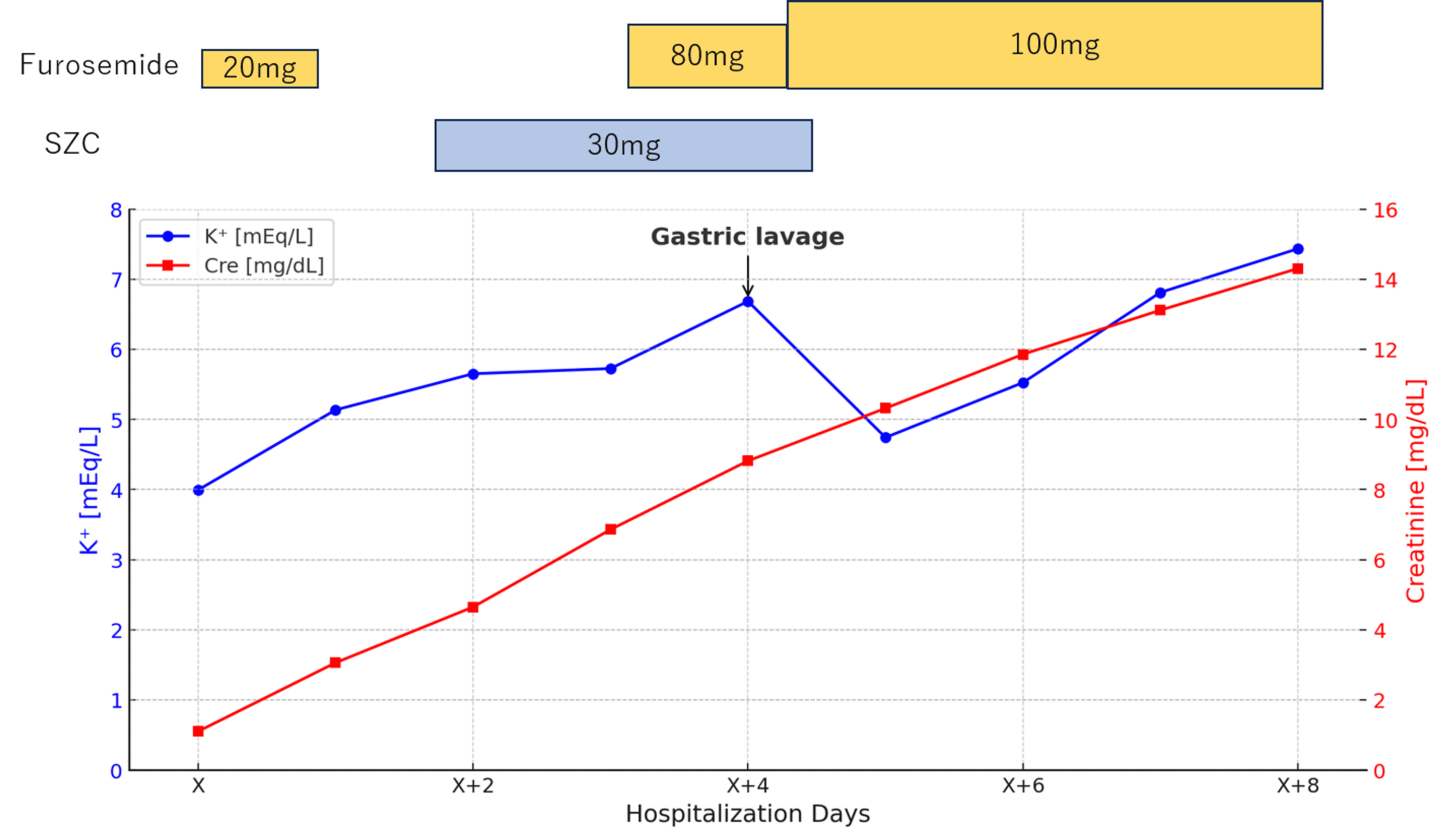
Serum potassium and creatinine levels before and after the administration of sodium zirconium cyclosilicate and furosemide K: potassium; Cre: creatinine; SZC: sodium zirconium cyclosilicate

Computed tomography scans revealed diffuse cerebral parenchymal swelling and blurring of the gray-white matter boundary in the brain, indicative of hypoxic encephalopathy. In the lungs, infiltrative shadows, thought to represent neurogenic pulmonary edema, were observed, and overall dilation of the intestines, which was considered shock bowel, was noted. On the same day, the patient was admitted to the EICU, and comprehensive management and hypothermia therapy were initiated. Owing to the expectation of a poor neurological prognosis, we decided not to perform further cardiopulmonary resuscitation or blood purification. After admission, dexmedetomidine at 0.2 μg/kg/h and fentanyl at 50 μg/h were continuously administered for sedation and analgesia, respectively. In addition, ampicillin/sulbactam administration was initiated because aspiration pneumonia was suspected. From day 2, worsening of renal function and increased serum potassium levels were observed, and treatment with SZC 30 mg/day was initiated. SZC was administered via a nasogastric tube, according to the method described in the interview form. On day 3, glucose-insulin therapy (500 mL of 10% dextrose with 10 units of insulin) was administered to control hyperkalemia; however, the effect was limited. Intravenous calcium chloride (5 mL of 8.5%) was also given on the same day. Thereafter, renal function continued to decline with ongoing oliguria, and potassium levels could not be controlled; therefore, furosemide 80 mg/day was started on day 4. The initial dose of SZC was continued from day 2 to day 5, and a total of 90 g was administered (Figure 1).

**Figure 2:**
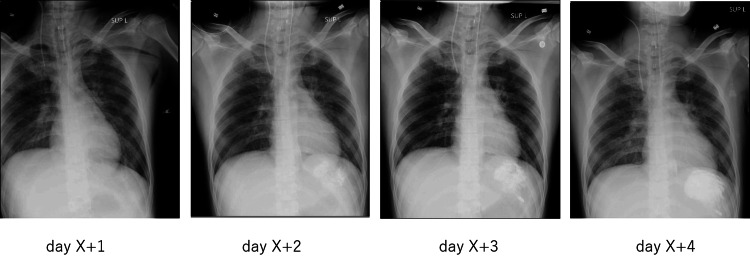
Changes in chest X-ray findings over time after admission The area of high radiopacity on radiography enlarged after the initiation of sodium zirconium cyclosilicate.

On day 4, GFO (glutamine, fiber, and oligosaccharide), a nutritional supplement commonly used in Japan to support gastrointestinal function and immunity, was administered enterally. On day 5, chest radiography revealed a high-density mass in the stomach. Daily chest radiography was performed for pulmonary follow-up, and a retrospective review confirmed the appearance of a high-density area in the stomach on day 2 that subsequently increased over time (Figure 2).

**Figure 3:**
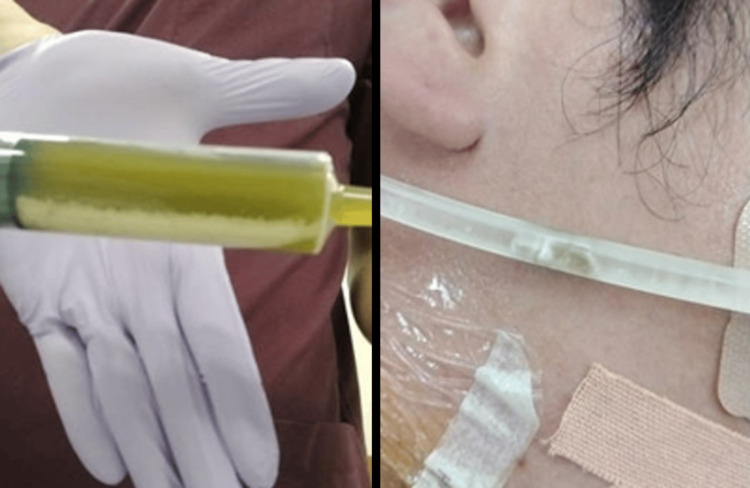
The aspiration of gastric contents through gastric lavage Gastric lavage was performed due to intragastric retention of sodium zirconium cyclosilicate.

The timing of SZC initiation and the appearance of radiographic findings coincided, strongly suggesting intragastric retention of SZC owing to precipitate formation. On the same day, a nasogastric tube was inserted, and aspiration of the gastric contents was attempted after injecting 100 mL of normal saline (Figure 3).

**Figure 4:**
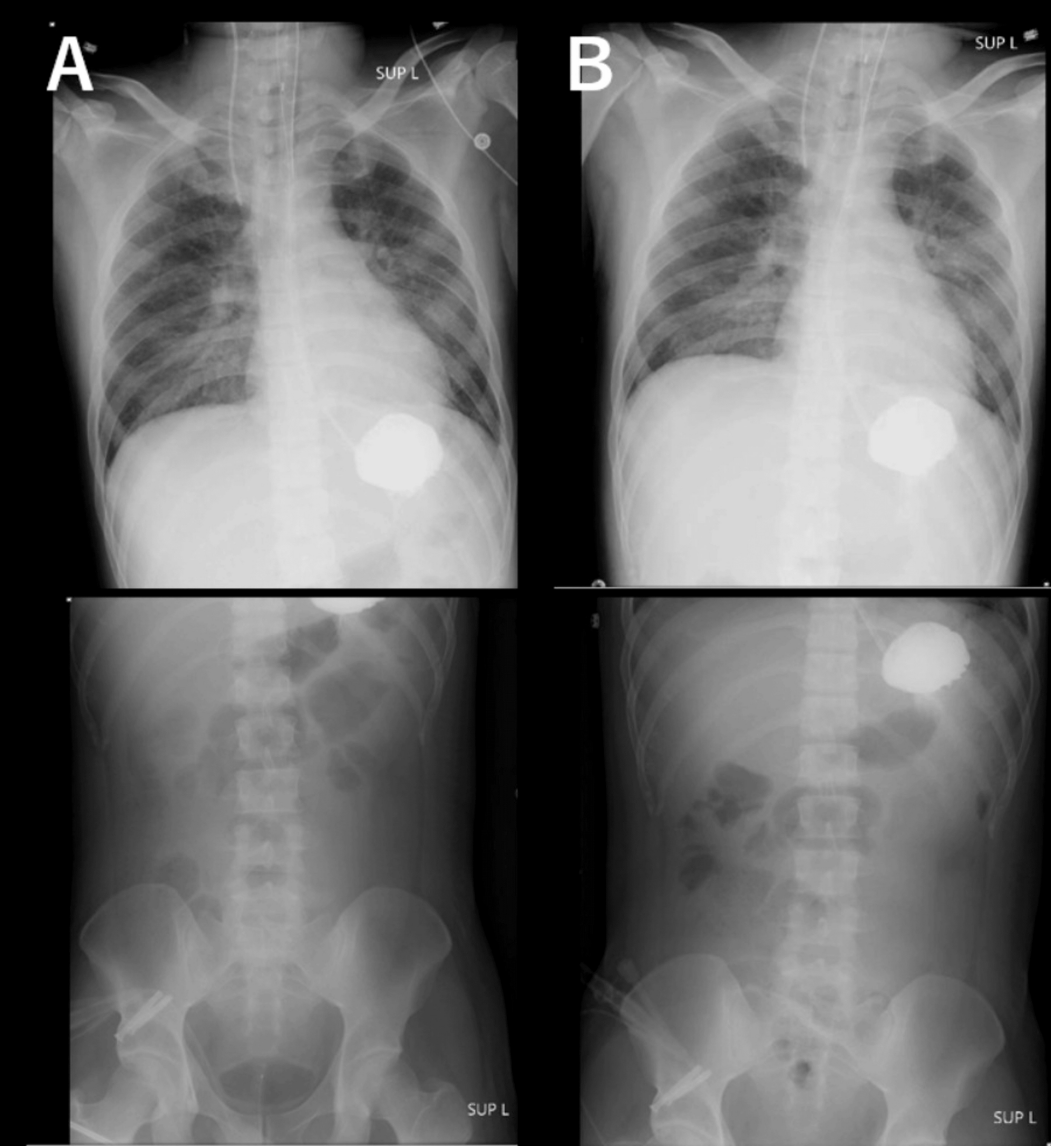
Post-procedure chest and abdominal X-rays A: X-rays taken immediately after gastric lavage and before Gastrografin administration. B: X-rays taken 12 h after Gastrografin administration.

A white precipitate and white solid material mixed with gastric juice were aspirated; however, as aspiration continued, the gastric juice became faintly bloody. The procedure was terminated, and gastric mucosal injury due to manipulation was suspected. A post-procedure chest radiograph showed a slight reduction in the high-density area of the stomach (Figure 4A).

**Figure 5:**
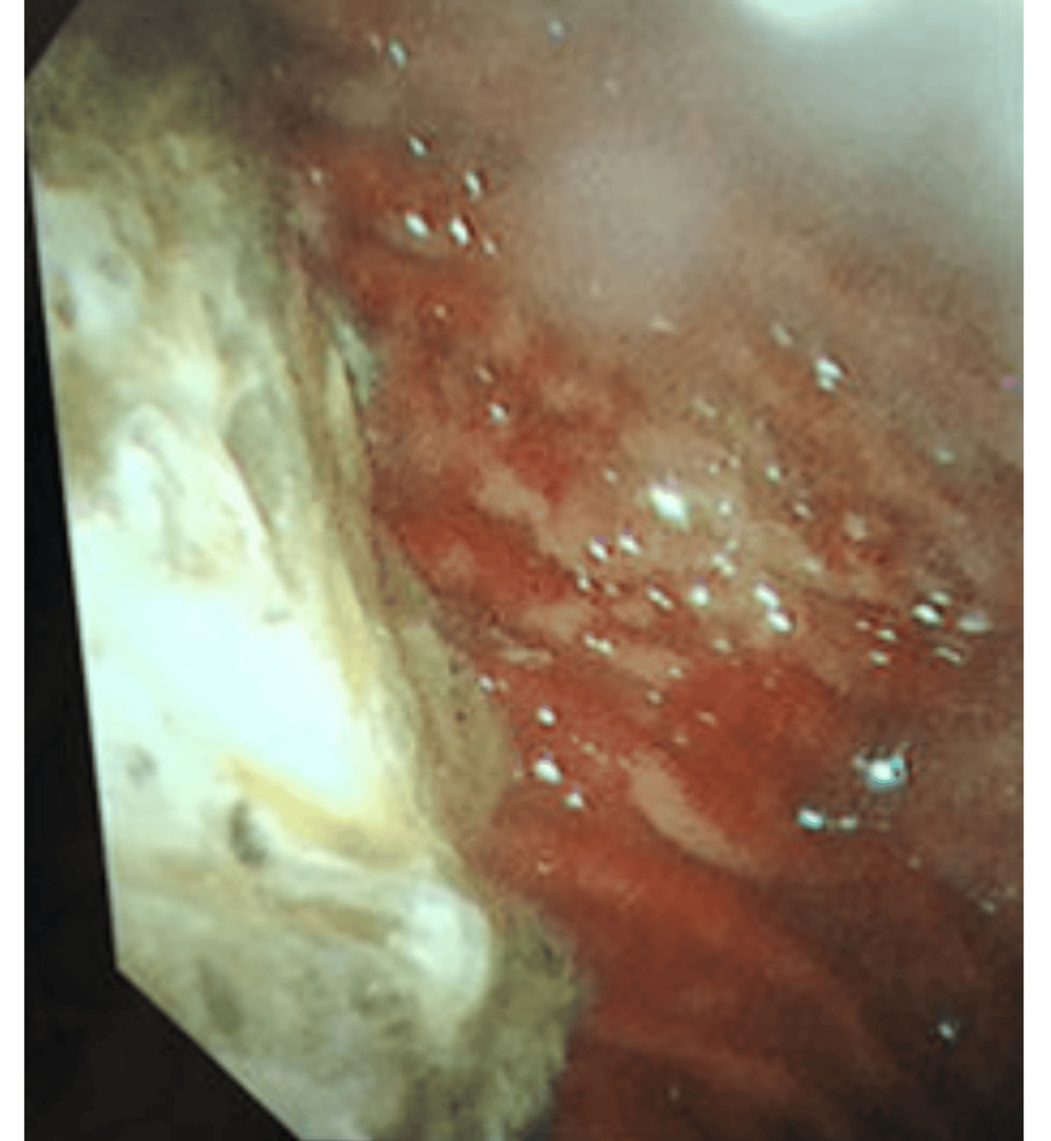
Sodium zirconium cyclosilicate precipitate was observed via endoscopy

Although some material was retrieved, most appeared to remain in the stomach. At this point, both SZC and GFO were discontinued. Subsequently, the serum potassium level temporarily decreased; however, with a further decline in renal function, it began to increase again. This suggested that a part of the SZC precipitate may have been expelled into the lower gastrointestinal tract during the gastric lavage procedure (Figure 1). Additionally, gastrografin was administered to assess gastric peristalsis, and follow-up radiography after 12 h showed that it remained in the stomach, suggesting severely impaired gastric motility (Figure 4B). On hospital day 6, gastric examination was performed using a bronchoscope, which revealed a white precipitate in the gastric fundus on gross examination (Figure 5).

On the same day, intravenous administration of metoclopramide hydrochloride 30 mg/day was started; however, there was no reduction in the high-density area in the stomach during the subsequent follow-up. Renal function later showed signs of improvement, with increased urine output and a trend toward decreased serum potassium levels; however, the patient died on day 13 due to respiratory failure. Subsequently, postcardiac arrest organ donation procedures were performed to harvest the eyes, heart valves, and major vessels.

## Discussion

The present case serves as a warning regarding the use of SZC, especially in patients with markedly reduced gastric motility, a condition frequently observed in critical illness, and is significant because of previously unreported side effects such as gastric retention and precipitate formation. It is important to acknowledge that the patient's critical status, including severe hypoxic encephalopathy, shock bowel, and the use of vasoactive and sedative medications, presents strong confounding factors that significantly contributed to the gastrointestinal hypomotility observed. SZC has demonstrated safety and effectiveness in numerous clinical trials. It was approved in Japan in 2020 and is widely used to treat hyperkalemia. Reports of side effects are few, with hypokalemia and congestive heart failure known as serious adverse effects; however, there have been no reports of gastric retention or precipitate formation. The retention and precipitate formation of SZC in the stomach involve the use of an insoluble powdered preparation, a severe decrease in bowel peristalsis due to intestinal ischemia following cardiac arrest, and the absence of bowel movements. In addition, sedatives, narcotic analgesics, and noradrenaline, which are used to maintain blood pressure, have been reported to suppress gastric peristalsis [4] and are believed to contribute to decreased intestinal peristalsis. With respect to patient positioning, the patient's posture was changed from right to left lateral decubitus every three to four hours; therefore, it was considered that it had little effect on the decreased gastrointestinal motility. Originally, this was a case of severe hyperkalemia that would have required blood dialysis; however, as the patient's neurological prognosis was poor, an active treatment approach was not pursued, and treatment with SZC was chosen. Regarding the method of administration, there are reports of safe administration via a nasogastric tube [5]; nasogastric tube administration was selected in the present case as well. In the present case, because the prognosis was extremely poor, the effects of gastric retention of SZC were limited to two points: persistent hyperkalemia and the inability to administer medication or enteral nutrition once the decline in gastric peristalsis became apparent. It has been reported that the potassium exchange capacity of SZC decreases in acidic environments with a low pH, such as the stomach [6]. In the present case, no decrease in the serum potassium levels was observed while SZC remained in the stomach. This suggests that the efficacy of SZC in lowering serum potassium depends on its passage beyond the stomach. The primary mechanism is thought to involve binding potassium excreted into the bowel rather than free potassium in the stomach. Loss of exchange capacity in precipitated SZC may also have contributed to the lack of effect. Other possible adverse events include changes in the absorption of other drugs caused by changes in gastric pH, nasogastric tube obstruction, a sudden decrease in serum potassium levels if the precipitate is evacuated at once, and the risk of intestinal obstruction or perforation due to the precipitate. There are reports of sigmoid colon perforation caused by fecalomas containing SZC in patients with decreased bowel peristalsis [7]. In the present case, owing to the background of intestinal ischemia and the possibility of mucosal damage, the risk of intestinal perforation was considered high, although obvious intestinal perforation did not occur. There are no previous reports on how to address gastric retention of SZC; however, in the present case, gastric lavage was performed, and agents that promote intestinal peristalsis were administered. After gastric lavage, radiographs showed a slight reduction in SZC precipitate, and there was a transient decrease in serum potassium level. The transient decrease in serum potassium following gastric lavage suggests that part of the SZC precipitate was expelled into the lower gastrointestinal tract, where it could effectively bind potassium ions. Although potassium binding by SZC is chemically reversible, its high selectivity and affinity make it stable and effective under physiological conditions. Thus, passage of the precipitate to the lower gastrointestinal tract remains the most plausible explanation for the transient potassium decrease. Destruction of the SZC gastric precipitate using endoscopy was also considered; however, active intervention was not undertaken in the present case; therefore, endoscopic fragmentation was not performed. If endoscopic fragmentation was possible, retrieval via a gastric tube or discharge into the lower gastrointestinal tract might also have been possible. However, in that situation, there is a risk of intestinal obstruction or hypokalemia; therefore, close observation is warranted. Currently, there are no established methods for managing gastric retention, and further case accumulation and evaluation are required. SZC is known to exhibit X-ray opacity and appears as a radiopaque bezoar-like mass. It has also been reported that this bezoar-like mass must be distinguished from gastrointestinal bleeding [8]; therefore, in the present case, endoscopic examination of the stomach was performed to rule out gastrointestinal bleeding. In this case, frequent radiographic examinations allowed the early recognition of gastric retention of SZC. Even in normal cases, SZC is radiopaque and appears as a bezoar-like mass. However, especially in patients with decreased intestinal peristalsis, monitoring the movement of SZC by radiographic examination may allow the early detection of gastric retention in severe cases.

## Conclusions

In the present case, we observed a rare and previously unreported adverse event of intragastric retention and SZC precipitate formation in a critically ill patient. Severe gastrointestinal hypomotility, potentially exacerbated by the patient's underlying critical illness, is highly suspected to be the key contributing factor to this phenomenon. Our findings suggest that, when administering SZC to patients with impaired gastric motility, particularly those critically ill, considering the risks of intragastric retention and precipitate formation is important. Given that this is a single case report with multiple confounding variables, further evidence is required to fully elucidate the causality and generalize this risk.
